# Assessing interstate racial and socioeconomic disparities in newborn screening policies in the United States

**DOI:** 10.3389/fpubh.2024.1310516

**Published:** 2024-04-29

**Authors:** Shanaya Bedford, Karl Vachuska

**Affiliations:** School of Medicine and Public Health and Department of Sociology, University of Wisconsin-Madison, Madison, WI, United States

**Keywords:** newborn screening policies, racial inequality, socioeconomic inequality, healthcare policy, state policy

## Abstract

**Introduction:**

This paper explores racial and socioeconomic disparities in newborn screening (NBS) policies across the United States. While inter-state inequality in healthcare policies is often considered a meaningful source of systemic inequity in healthcare outcomes, to the best of our knowledge, no research has explored racial and socioeconomic disparities in newborn screening policies based on state of residence.

**Methods:**

We investigate these disparities by calculating weighted average exposure to specific NBS tests by racial and socioeconomic group. We additionally estimate count models of the number (and type) of NBS conditions screened for by state racial and socioeconomic composition.

**Results:**

Adding to the knowledge base that social determinants of health and health disparities are linked, our analysis surprisingly reveals little evidence of substantial inter-state inequity in newborn screenings along racial and socioeconomic lines.

**Discussion:**

While there is substantial nationwide racial and socioeconomic inequity in terms of infant health, the distribution of state-level policies does not appear to be structured in a manner to be a driver of these disparities. Our findings suggest that efforts to reduce inequities in outcomes related to NBS should shift focus toward the delivery of screening results and follow-up care as discussion builds on expanding NBS to include more conditions and genomic testing.

## Introduction

It is commonly acknowledged and accepted that social determinants of health drive health disparities, which worsen health outcomes for disadvantaged populations (1, 2). Many social determinants, such as economic status, education, and physical environments, vary greatly based on inequities driven by race and ethnicity (3). Infant mortality is considered one of the most prominent inequities and measures of our healthcare system. The infant mortality rate of Non-Hispanic Black infants is 2.4 times that of White infants in the United States (4). Racial disparities in infant mortality underscore the need for greater research on racial and socioeconomic inequalities in newborn screening policies in the United States (5).

In the United States, a newborn screening is performed on essentially every newborn. Newborn screenings entail a screening of specific rare metabolic, endocrine, and genetic disorders that require early detection and intervention to prevent serious health problems, disabilities, or even death (6). The blood spots used to test for these conditions must be collected 24–48 h after birth in order to minimize false negatives and promptly notify caregivers in case of positive results (7). Furthermore, a hearing test and a test for critical congenital heart disease are also conducted (8). Newborn screenings are set at the state level, and thus, we determined it as the geographical unit of analysis through which possible disparities from the newborn screening policies must be analyzed. Each state determines the conditions it screens for based on its population and also decides the cutoffs for positive results. Within the last 20 years, the Recommended Uniform Screening Panel (RUSP) was created by the Advisory Committee on Heritable Disorders in Newborns and Children and the Secretary of the Department of Health and Human Services (HHS) to act as a national framework of NBS conditions, but states are free to omit these conditions or include additional ones depending on stakeholders within a state and state legislature (9).

RUSP helps to ensure equitable practices, but questions about which states will implement screening for newly added conditions arise as the RUSP gets updated to reflect new technologies and available treatments. Although the cost of newborn screenings differs by state, it is generally covered by insurance and included in the birthing charges. In the case of families that are eligible, CHIP or Medicaid can pay for the cost of the newborn screening (10). While the federal government has attempted to implement a nationwide standard of conditions to screen for, it is ultimately the state public health departments that determine the set of conditions for their population. Therefore, there is substantial variation between states in terms of the conditions screened. State-level policies have been highlighted in the past as a source of inequity in healthcare in the United States (11, 12). State-level healthcare policies have the ability to either amplify or attenuate nationwide racial and socioeconomic inequities in terms of healthcare. In spite of the state level being the key level of government in newborn screening policies, there has not been adequate research (to our knowledge) conducted to explore nationwide inequities in exposure to newborn screening policies as a result of state residence. This might be because the newborn screening is considered one of the greatest public health achievements, and the RUSP and state-level discretion of conditions screened is supposed to be representative of a region’s population, which should account for conditions diagnosed more frequently in certain populations and the therapeutic options available.

This study aims to contribute to the public health scholarship surrounding newborn screening by exploring racial and socioeconomic inequity in newborn screening policies by state due to the lack of literature on state-to-state comparisons for NBS practices and outcomes. Our research question looks to identify inter-state racial or socioeconomic inequities in NBS.

## Methods

### Study design

Using a cross-sectional dataset on state NBS policies, we explore racial and socioeconomic disparities by calculating weighted average exposure to specific NBS tests by state of residence. To explore larger trends in policy counts, we also estimate count models of the number (and type) of NBS conditions screened for by state racial and socioeconomic composition.

### Data

Two sources were used to procure data for this project: babysfirsttest.com and the American Community Survey 2021 1-year estimates. Baby’s First Test is a website that serves as a national “newborn screening education resource center for families and health professionals” (13). Baby’s First Test provides a unique assortment of information on newborn screening, including state-specific information about newborn screening programs and condition-specific information. Various authorative and administrative sources, such as health agencies, advocacy organizations, and public health programs, contribute to the website’s information. The site undergoes ongoing review and updates to ensure accuracy. We drew on Baby’s First Test in order to access a current (2023) list of newborn screening tests performed in each state.

The American Community Survey (ACS) is a monthly survey run by the U.S. Census Bureau to obtain detailed demographic data at various geographical resolutions about the American population. The ACS aims for a 1% yearly random sample of households in all 50 states and the District of Columbia. We obtained 2021 ACS data from the Social Explorer data platform. Specifically, we obtained poverty rate and racial composition for 2021 (the most recent year available) at the state level for all 50 states and the District of Columbia. We defined individuals as “below poverty” or “above poverty” based on statistics from the 2021 ACS. The U.S. Census Bureau determines poverty status based on income thresholds specific to family size (14). This information is obtained specifically from U.S. Census Bureau 2021 1-year estimates table B17001.

### Statistical analyses

Using the two aforementioned data sources, a dataset was constructed to contain the disorders tested for in each state. For each disorder, we estimated group-level residence in states that tested for each disorder. For example, for a particular disorder *d*, we calculated the proportion of Black Americans that resided in states that tested for that disorder using the following formula:


$$p_{d}=\frac{\sum B_{i}}{\sum B_{i}+\sum B_{j}}$$

Where i refers to a vector of states that test for disorder d, and j refers to a vector of states that do NOT test for disorder d. Additionally, $B_{i}$ represents the number of Black individuals who reside in state i.

The study included data from all 50 states (and the District of Columbia), and information was collected pertaining to the count of disorders tested for, poverty rates, and racial composition from the American Community Survey (ACS) 2021 1-year estimates. Poisson regression models were subsequently used to analyze the data and explore the relationship between the count of disorders tested for and state racial and socioeconomic composition.

Three models were constructed to investigate the influence of different predictors on the count of disorders tested in each state. Model 1 included only the poverty rate as the predictor variable. Model 2 incorporated predictors related to racial composition. Model 3 included both poverty and racial composition variables.

The formula for Model 3 can be represented as follows:


$$\begin{array}{c} \ln\left(\mu \right)=\beta 0+\beta 1*\mathrm{prop}\_\mathrm{poor}+\beta 2*\mathrm{prop}\_\mathrm{black}\\ +\beta 3*\mathrm{prop}\_\mathrm{hispanic}+\beta 4*\mathrm{prop}\_\mathrm{asian}\\ +\beta 5*\mathrm{prop}\_\mathrm{native}+\beta 6*\mathrm{prop}\_\mathrm{other} \end{array}$$

We additionally explored the count of disorders tested based on five more specific categories of disorders: Amino Acid Disorders, Fatty Acid Oxidation Disorders, Hemoglobin Disorders, Lysosomal Storage Disorders, and all other disorders.

We evaluated the fit of all models based on AIC, BIC, and adjusted R-squared variables. We particularly drew on adjusted R-squared as a means for understanding to what extent our sets of variables were meaningful in explaining variance between states in the count of disorders tested for.

## Results

### Overall rates

Table 1 presents the proportions of individuals across various groups who receive newborn screenings for various conditions based on their state of residence. From an analysis of the table, it is apparent that there is some variation in the screening rates across population groups for certain conditions. In terms of Argininosuccinic Aciduria (ASA), Citrullinemia (Type I), Classic Phenylketonuria (PKU), Homocystinuria (HCY), as well as a few other conditions, all states screen for these conditions, as denoted by a value of 1.000 across all groups.

**Table 1:** Weighted average screening exposure by screening test and racial/socioeconomic categories.

	All	Above poverty	Below poverty	White	Black	Hispanic	Asian	Native	Other
Argininemia (ARG)	0.717	0.717	0.714	0.709	0.628	0.769	0.814	0.635	0.710
Argininosuccinic Aciduria (ASA)	1.000	1.000	1.000	1.000	1.000	1.000	1.000	1.000	1.000
Benign Hyperphenylalaninemia (H-PHE)	0.772	0.772	0.776	0.745	0.760	0.852	0.840	0.688	0.751
Biopterin Defect in Cofactor Biosynthesis (BIOPT-BS)	0.604	0.604	0.604	0.577	0.577	0.704	0.650	0.426	0.573
Biopterin Defect in Cofactor Regeneration (BIOPT-REG)	0.594	0.594	0.597	0.563	0.577	0.696	0.646	0.415	0.562
Citrullinemia, Type I (CIT)	1.000	1.000	1.000	1.000	1.000	1.000	1.000	1.000	1.000
Citrullinemia, Type II (CIT II)	0.820	0.821	0.814	0.808	0.756	0.881	0.886	0.723	0.812
Classic Phenylketonuria (PKU)	1.000	1.000	1.000	1.000	1.000	1.000	1.000	1.000	1.000
Homocystinuria (HCY)	1.000	1.000	1.000	1.000	1.000	1.000	1.000	1.000	1.000
Hypermethioninemia (MET)	0.783	0.783	0.786	0.764	0.747	0.856	0.843	0.641	0.761
Maple Syrup Urine Disease (MSUD)	1.000	1.000	1.000	1.000	1.000	1.000	1.000	1.000	1.000
Tyrosinemia, Type I (TYR I)	1.000	1.000	1.000	1.000	1.000	1.000	1.000	1.000	1.000
Tyrosinemia, Type II (TYR II)	0.807	0.806	0.811	0.803	0.763	0.856	0.811	0.722	0.776
Tyrosinemia, Type III (TYR III)	0.791	0.791	0.791	0.786	0.760	0.836	0.806	0.561	0.760
Congenital Adrenal Hyperplasia (CAH)	1.000	1.000	1.000	1.000	1.000	1.000	1.000	1.000	1.000
Primary Congenital Hypothyroidism (CH)	1.000	1.000	1.000	1.000	1.000	1.000	1.000	1.000	1.000
2,4 Dienoyl-CoA Reductase Deficiency (DE RED)	0.459	0.457	0.466	0.477	0.511	0.407	0.392	0.198	0.416
Carnitine Acylcarnitine Translocase Deficiency (CACT)	0.852	0.851	0.860	0.840	0.840	0.894	0.879	0.732	0.834
Carnitine Palmitoyltransferase I Deficiency (CPT-IA)	0.768	0.768	0.765	0.744	0.709	0.864	0.840	0.661	0.751
Carnitine Palmitoyltransferase Type II Deficiency (CPT-II)	0.861	0.860	0.869	0.855	0.842	0.896	0.881	0.733	0.842
Carnitine Uptake Defect (CUD)	1.000	1.000	1.000	1.000	1.000	1.000	1.000	1.000	1.000
Glutaric Acidemia, Type II (GA-2)	0.847	0.848	0.848	0.841	0.805	0.892	0.877	0.723	0.830
Long-Chain L-3 Hydroxyacyl-CoA Dehydrogenase Deficiency (LCHAD)	1.000	1.000	1.000	1.000	1.000	1.000	1.000	1.000	1.000
Medium-Chain Acyl-CoA Dehydrogenase Deficiency (MCAD)	1.000	1.000	1.000	1.000	1.000	1.000	1.000	1.000	1.000
Medium-Chain Ketoacyl-CoA Thiolase Deficiency (MCAT)	0.442	0.441	0.448	0.457	0.482	0.400	0.385	0.370	0.412
Medium/Short-Chain L-3 Hydroxyacyl-CoA Dehydrogenase Deficiency (M/SCHAD)	0.611	0.612	0.608	0.581	0.602	0.696	0.742	0.281	0.561
Short-Chain Acyl-CoA Dehydrogenase Deficiency (SCAD)	0.698	0.697	0.706	0.662	0.660	0.823	0.748	0.641	0.674
Trifunctional Protein Deficiency (TFP)	1.000	1.000	1.000	1.000	1.000	1.000	1.000	1.000	1.000
Very Long-Chain Acyl-CoA Dehydrogenase Deficiency (VLCAD)	1.000	1.000	1.000	1.000	1.000	1.000	1.000	1.000	1.000
Hemoglobinopathies (Var Hb)	0.882	0.880	0.898	0.865	0.930	0.900	0.919	0.747	0.876
S, Beta-Thalassemia (Hb S/ÃŸTh)	0.985	0.985	0.986	0.981	0.998	0.990	0.990	0.983	0.980
S, C Disease (Hb S/C)	0.985	0.985	0.986	0.981	0.998	0.990	0.990	0.983	0.980
Sickle Cell Anemia (Hb SS)	1.000	1.000	1.000	1.000	1.000	1.000	1.000	1.000	1.000
Organic Acid Conditions	1.000	1.000	1.000	1.000	1.000	1.000	1.000	1.000	1.000
2-Methyl-3-Hydroxybutyric Acidemia (2M3HBA)	0.711	0.710	0.715	0.687	0.665	0.806	0.767	0.644	0.681
2-Methylbutyrylglycinuria (2MBG)	0.798	0.797	0.806	0.781	0.788	0.860	0.828	0.692	0.763
3-Hydroxy-3-Methylglutaric Aciduria (HMG)	1.000	1.000	1.000	1.000	1.000	1.000	1.000	1.000	1.000
3-Methylcrotonyl-CoA Carboxylase Deficiency (3-MCC)	0.972	0.971	0.978	0.973	0.992	0.981	0.934	0.964	0.937
3-Methylglutaconic Aciduria (3MGA)	0.766	0.767	0.764	0.745	0.714	0.863	0.815	0.662	0.729
Beta-Ketothiolase Deficiency (BKT)	1.000	1.000	1.000	1.000	1.000	1.000	1.000	1.000	1.000
Glutaric Acidemia, Type I (GA-1)	1.000	1.000	1.000	1.000	1.000	1.000	1.000	1.000	1.000
Holocarboxylase Synthetase Deficiency (MCD)	0.979	0.979	0.981	0.973	0.985	0.992	0.991	0.995	0.982
Isobutyrylglycinuria (IBG)	0.734	0.732	0.745	0.711	0.694	0.817	0.786	0.645	0.728
Isovaleric Acidemia (IVA)	1.000	1.000	1.000	1.000	1.000	1.000	1.000	1.000	1.000
Malonic Acidemia (MAL)	0.680	0.680	0.679	0.655	0.619	0.763	0.798	0.630	0.673
Methylmalonic Acidemia (CobalaminDisorders) (Cbl A,B)	0.976	0.976	0.979	0.972	0.989	0.980	0.981	0.981	0.972
Methylmalonic Acidemia (Methymalonyl-CoA Mutase Deficiency) (MUT)	0.989	0.989	0.991	0.988	0.991	0.990	0.991	0.998	0.989
Methylmalonic Acidemia with Homocystinuria (Cbl C, D, F)	0.825	0.825	0.826	0.811	0.801	0.881	0.864	0.705	0.804
Propionic Acidemia (PROP)	1.000	1.000	1.000	1.000	1.000	1.000	1.000	1.000	1.000
Adrenoleukodystrophy (ALD)	0.803	0.804	0.797	0.777	0.788	0.885	0.868	0.642	0.781
Biotinidase Deficiency (BIOT)	1.000	1.000	1.000	1.000	1.000	1.000	1.000	1.000	1.000
Classic Galactosemia (GALT)	1.000	1.000	1.000	1.000	1.000	1.000	1.000	1.000	1.000
Critical Congenital Heart Disease (CCHD)	0.985	0.984	0.985	0.980	0.998	0.990	0.989	0.943	0.980
Cystic Fibrosis (*CF*)	1.000	1.000	1.000	1.000	1.000	1.000	1.000	1.000	1.000
Galactoepimerase Deficiency (GALE)	0.230	0.231	0.223	0.254	0.286	0.135	0.184	0.261	0.231
Galactokinase Deficiency (GALK)	0.230	0.231	0.223	0.254	0.286	0.135	0.184	0.261	0.231
Hearing loss (HEAR)	0.975	0.974	0.976	0.966	0.997	0.985	0.987	0.933	0.970
Severe Combined Immunodeficiency (SCID)	1.000	1.000	1.000	1.000	1.000	1.000	1.000	1.000	1.000
Spinal Muscular Atrophy (SMA)	0.869	0.870	0.860	0.865	0.843	0.904	0.871	0.846	0.849
T-cell Related Lymphocyte Deficiencies	0.483	0.482	0.496	0.408	0.463	0.688	0.661	0.172	0.458
Carbamoyl Phosphate Synthetase I Deficiency (CPS)	0.252	0.253	0.248	0.206	0.216	0.380	0.389	0.084	0.249
Hyperornithine with Gyrate Deficiency (Hyper ORN)	0.166	0.168	0.159	0.128	0.099	0.275	0.346	0.063	0.172
Ornithine Transcarbamylase Deficiency (OTC)	0.241	0.243	0.232	0.196	0.182	0.377	0.386	0.077	0.240
Prolinemia (PRO)	0.128	0.130	0.121	0.083	0.054	0.260	0.315	0.066	0.130
Mucopolysaccharidosis Type-I (MPS I)	0.773	0.776	0.754	0.786	0.799	0.704	0.829	0.514	0.782
Pompe (POMPE)	0.795	0.798	0.771	0.817	0.809	0.712	0.840	0.532	0.800
Ethylmalonic Encephalopathy (EME)	0.227	0.228	0.223	0.174	0.197	0.369	0.377	0.077	0.223
Formiminoglutamic Acidemia (FIGLU)	0.150	0.152	0.140	0.106	0.073	0.276	0.346	0.060	0.157
Hyperornithinemia-Hyperammonemia-Homocitrullinuria Syndrome (HHH)	0.175	0.176	0.172	0.136	0.126	0.277	0.348	0.070	0.179
Human Immunodeficiency Virus (HIV)	0.071	0.070	0.074	0.067	0.077	0.072	0.100	0.022	0.068
Glucose-6-Phosphate Dehydrogenase Deficiency (G6PD)	0.002	0.002	0.003	0.001	0.007	0.001	0.001	0.000	0.002
Pyroglutamic Acidemia (5-OXO)	0.049	0.049	0.052	0.048	0.078	0.039	0.041	0.014	0.039
Krabbe	0.307	0.305	0.311	0.340	0.360	0.195	0.266	0.074	0.283
Fabry (FABRY)	0.137	0.139	0.127	0.146	0.164	0.100	0.131	0.045	0.132
Gaucher (GBA)	0.118	0.120	0.112	0.130	0.118	0.089	0.109	0.040	0.112
Mucopolysaccharidosis Type-II (MPS II)	0.057	0.057	0.055	0.063	0.060	0.041	0.045	0.015	0.052
Niemann-Pick Disease (NPD)	0.066	0.067	0.059	0.064	0.072	0.068	0.087	0.011	0.052
Nonketotic Hyperglycinemia (NKH)	0.035	0.034	0.040	0.045	0.035	0.010	0.010	0.009	0.032
Congenital Toxoplasmosis (TOXO)	0.025	0.026	0.019	0.031	0.012	0.015	0.028	0.003	0.030
Guanidinoacetate Methyltransferase Deficiency (GAMT)	0.070	0.069	0.072	0.068	0.068	0.070	0.095	0.032	0.068
Congenital Cytomegalovirus	0.010	0.011	0.007	0.013	0.001	0.008	0.004	0.011	0.010

Mild differences are observed when considering many other conditions, such as Benign Hyperphenylalaninemia (H-PHE), Biopterin Defect in Cofactor Biosynthesis (BIOPT-BS), and Biopterin Defect in Cofactor Regeneration (BIOPT-REG). In these cases, variation along racial and socioeconomic lines becomes evident. For example, in Argininemia (ARG), the screening rate for White, Hispanic, and Asian individuals is considerably higher at 0.709, 0.769, and 0.814 compared to Black individuals at 0.628.

For 2,4 Dienoyl-CoA Reductase Deficiency (DE RED), while nearly half of White Americans reside in states that test for the condition, only 19.8% of Native Americans do. Several disorders appear to have substantial disparities in testing between White and Native Americans. Specific racial differences are also apparent when examining the data on hemoglobinopathies, including Sickle Cell Anemia (Hb SS) and other related conditions. The screening rate for Black individuals is consistently equal or higher, compared to White individuals, across all hemoglobinopathies. This potentially indicates a targeted approach to screening for these conditions disproportionately in the Black population.

### Overall number of disorders

Table 2 presents the results of Poisson regression models, which were employed to predict the total count of all disorders tested in each state. Model 1 includes only the poverty rate, which has a coefficient of 0.50 and is not statistically significant. In Model 2, the racial composition variables are included as predictors. The coefficient for the percentage of the population that is black is 0.43* (*p* < 0.05), indicating a positive association with the count of disorders tested. Additionally, the coefficient for the percentage of the population that is Asian is statistically significant at 1.78* (*p* < 0.05), and the coefficient for the percentage of the population that is “Other” is statistically significant at-2.68* (*p* < 0.05). Model 3 includes racial composition variables alongside the poverty rate. Among these predictor variables, only the coefficient for the percentage of the population that is “Other” is statistically significant at-2.64* (*p* < 0.05). Sensitivity analysis reveals that this significant finding is entirely dependent on the inclusion of “Hawaii” in the sample, the state with the highest proportion of “Other individuals (29%) and a below-average number of disorders tested (44).

**Table 2:** Poisson model predicting total number of disorders screened for by state.

	Model 1	Model 2	Model 3
Prop. poverty	0.50 (0.73)		−0.08 (0.98)
Prop. Black		0.43 * (0.20)	0.44 (0.26)
Prop. Hispanic		0.01 (0.24)	0.02 (0.25)
Prop. Asian		1.78 * (0.88)	1.75 (0.96)
Prop. Native		0.21 (0.95)	0.21 (0.96)
Prop. other		−2.68 * (1.25)	−2.64 * (1.33)
N	51	51	51
AIC	412.24	406.17	408.16
BIC	416.10	417.76	421.68
Adj. R2	0.01	0.25	0.25

***p < 0.001; **p < 0.01; *p < 0.05.

In terms of other non-White racial groups, the proportion Black, proportion Hispanic, proportion Native, and proportion Asian coefficients are all positive in Model 3. None of these coefficients are statistically significant, however. The adjusted R-squared indicates that 25% of the variability in the count of disorders tested can be explained by the predictors in Models 2 and 3 and subsequently suggests that racial composition has some appreciable predictive power. AIC scores indicate that Model 2 is the best fit, while BIC scores indicate that Model 1 is the best fit. In conclusion, there seems to be no significant association between poverty rates and the count of disorders tested. Some of the racial composition variables exhibit statistically significant associations, but generally not in the direction one would expect.

### Amino acid disorders

Table 3 presents the results of Poisson regression models that specifically focus on amino acid disorders. The table is structured like Table 2, with Model 1, Model 2, and Model 3 including poverty, racial composition, and both poverty and racial composition variables, respectively. Across all three models, none of the variables are significant. Adjusted R-squared values suggest poverty can explain virtually no variance in the count of amino acid disorders tested for, and racial composition variables can only explain approximately 7% of the variance. AIC and BIC indicate best fit in Model 1 (the poverty model), suggesting that while racial composition can explain some variance, neither model is very parsimonious. Furthermore, the finding that the poverty rate, as well as all but one non-White racial group, is positively associated with the count of amino acid disorders tested for suggests that there is no traditional racial or socioeconomic inequity in exposure to amino acid disorder testing.

**Table 3:** Poisson model predicting number of amino acid disorders screened for by state.

	Model 1	Model 2	Model 3
Prop. poverty	0.34 (1.54)		0.19 (2.08)
Prop. Black		0.31 (0.43)	0.28 (0.55)
Prop. Hispanic		0.05 (0.49)	0.03 (0.53)
Prop. Asian		2.27 (1.84)	2.34 (1.99)
Prop. Native		0.34 (2.02)	0.33 (2.02)
Prop. other		−3.37 (2.63)	−3.45 (2.78)
N	51	51	51
AIC	279.26	283.38	285.37
BIC	283.13	294.97	298.89
Adj. R2	0.00	0.07	0.07

***p < 0.001; **p < 0.01; *p < 0.05.

### Fatty acid oxidation disorders

Table 4 presents the results of Poisson regression models focusing specifically on fatty acid oxidation disorders. Across all three models, only two predictors are positive (proportion other in Models 2 and 3; proportion Hispanic in Model 3). No variables are significant. Furthermore, adjusted R-squared values are very low across all three models, suggesting that racial and socioeconomic variables explain little variation in the count of fatty acid oxidation disorders tested by state. In fact, the finding that the poverty rate, as well as all but two non-White racial groups, are positively associated with the number of fatty acid oxidation disorders tested for suggests little evidence to support the existence of racial or socioeconomic inequity in exposure to fatty acid oxidation disorder testing in traditional terms.

**Table 4:** Poisson model predicting number of fatty acid oxidation disorders screened for by state.

	Model 1	Model 2	Model 3
Prop. poverty	1.46 (1.66)		0.98 (2.24)
Prop. Black		0.67 (0.46)	0.51 (0.59)
Prop. Hispanic		0.00 (0.54)	−0.10 (0.58)
Prop. Asian		2.31 (2.01)	2.68 (2.18)
Prop. Native		0.95 (2.17)	0.91 (2.18)
Prop. other		−3.18 (2.86)	−3.63 (3.02)
N	51	51	51
AIC	258.75	263.00	264.81
BIC	262.62	274.59	278.33
Adj. R2	0.02	0.09	0.09

***p < 0.001; **p < 0.01; *p < 0.05.

### Hemoglobin disorders

Table 5 presents the results of Poisson regression models focusing specifically on hemoglobin disorders. Research indicates substantial racial inequity in the risk of having a hemoglobin disorder. Sickle cell trait is especially more common in Black Americans compared to White Americans (15). As such, observing that states with more Black residents test for fewer hemoglobin disorders would be especially concerning in terms of health equity.

**Table 5:** Poisson model predicting number of hemoglobin disorders screened for by state.

	Model 1	Model 2	Model 3
Prop. poverty	1.13 (1.24)		0.56 (1.68)
Prop. Black		0.40 (0.35)	0.31 (0.45)
Prop. Hispanic		0.18 (0.39)	0.13 (0.43)
Prop. Asian		0.64 (1.51)	0.86 (1.65)
Prop. Native		1.16 (1.56)	1.14 (1.57)
Prop. other		−1.00 (2.15)	−1.26 (2.28)
N	51	51	51
AIC	267.20	273.66	275.54
BIC	271.06	285.25	289.07
Adj. R2	0.02	0.05	0.05

***p < 0.001; **p < 0.01; *p < 0.05.

Among all three models, none of the coefficients reach statistical significance, suggesting that no socioeconomic or racial composition variables are significantly associated with the count of hemoglobin disorders tested for across states. Furthermore, adjusted R-squared variables were low across all three models—suggesting state racial and socioeconomic composition variables are not important for explaining variation in the count of hemoglobin disorders tested by state. Ultimately, it is especially crucial that we did not observe substantial inequalities between Black and White Americans in terms of hemoglobin disorder testing, as evidence suggests Black Americans may be at greater risk of having a hemoglobin disorder.

### Lysosomal storage disorders

Table 6 presents the results of Poisson regression models focused on lysosomal storage disorders. Model 1 suggests that poverty is not statistically associated with the count of disorders tested. Notably, the coefficient for proportion Native American is negative and highly significant (*p* < 0.001) in Model 2, indicating a negative association between the percentage of the population that is Native American and the count of lysosomal storage disorders tested. Sensitivity analysis reveals that removing Montana, North Dakota, or South Dakota from the sample attenuates this coefficient to marginal significance (*p* < 0.1). Model 3 expands on the racial composition variables by also adding in the poverty rate. The inclusion of the poverty rate does not substantially alter the coefficient for proportion Native American. Notably, among all categories of disorders we examined, racial composition variables best explained variance in the count of disorders tested (for lysomal storage disorders) as indicated by adjusted R-squared values of 0.37 and 0.38 in Models 2 and 3, respectively. This suggests that state racial composition variables may, in particular, be important variables for understanding variance in how many lysomal storage disorders states test for.

**Table 6:** Poisson model predicting number of lysomal disorders screened for by state.

	Model 1	Model 2	Model 3
Prop. poverty	−4.56 (3.94)		−5.44 (5.09)
Prop. Black		0.11 (1.01)	0.97 (1.29)
Prop. Hispanic		−1.27 (1.49)	−0.91 (1.53)
Prop. Asian		6.87 (4.65)	5.11 (4.95)
Prop. Native		−28.39 * (12.53)	−27.52 * (12.57)
Prop. other		−14.12 (7.25)	−12.17 (7.61)
N	51	51	51
AIC	194.97	181.57	182.40
BIC	198.83	193.16	195.92
Adj. R2	0.03	0.37	0.38

***p < 0.001; **p < 0.01; *p < 0.05.

To summarize these specific findings, the Poisson regression models focusing on lysosomal storage disorders indicate a lack of statistical significance for both the poverty rate and the majority of racial composition variables. However, the percentage of the population that is Native American has demonstrated a significant negative association with the count of disorders tested for in newborn screening programs. While this potentially suggests an axis of racial inequity in newborn screening policy, it is important to note that this finding appears sensitive to the inclusion of outliers. Additionally, since limited research has been done on the genetic conditions of the Native American population, it is also difficult to theorize about the implications of such an inequity.

### Other disorders

Table 7 presents the results of Poisson regression models focused on all other disorders, excluding the specific disorder categories exhibited in the tables listed above. Across all three models, a number of variables are negatively associated with the count of other disorders tested for (proportion in poverty in Models 1 and 3, proportion Hispanic in Model 2, proportion Native American in Models 2 and 3, proportion other in Models 2 and 3). While a negative association with these variables would align with traditional axes of racial and socioeconomic inequity, no coefficient is statistically significant.

**Table 7:** Poisson model predicting number of other disorders screened for by state.

	Model 1	Model 2	Model 3
Prop. poverty	−0.39 (1.62)		−1.34 (2.18)
Prop. Black		0.36 (0.45)	0.58 (0.57)
Prop. Hispanic		−0.09 (0.53)	0.04 (0.57)
Prop. Asian		1.60 (1.95)	1.09 (2.12)
Prop. Native		−0.85 (2.16)	−0.78 (2.15)
Prop. other		−2.41 (2.77)	−1.80 (2.95)
N	51	51	51
AIC	235.17	240.00	241.61
BIC	239.04	251.59	255.14
Adj. R2	0.00	0.06	0.07

***p < 0.001; **p < 0.01; *p < 0.05.

## Discussion

In this paper, inter-state variation in newborn screening policies was analyzed with a specific focus on how different racial and socioeconomic groups are subject to different newborn screening policies. Our results show little evidence of substantial inter-state racial or socioeconomic inequity in newborn screenings. This is in contrast to a wide breadth of public health research, which has established that lower-income and non-White individuals disproportionately live in lower-resourced areas (16–19). Analyses of specific conditions found significant disparities for a small number of conditions, but few at the expense of non-White or impoverished Americans. We did, however, observe states with a larger “Other” (Pacific-Islander or multiracial) population screen for a significantly lower number of disorders overall. We additionally found that states with a larger Native American population screen for a significantly lower number of Lysosomal storage disorders. This is a notable finding because Lysosomal storage disorders, such as Pompe disease and Mucopolysaccharidosis type I, which were recently added to the RUSP, are screened for by two methods, “mass spectrometry (MS/MS) and digital microfluidics fluorimetry (DMF-F),” with MS/MS providing better precision (20, 21). Lysosomal storage disorders are generally screened for by analyzing enzymatic activity from the dried blood spots collected for the NBS, but this method frequently returns high false positives, which require follow-up testing and care required to achieve accurate results (20). This potentially adds to the extensive history of Native American populations experiencing healthcare disparities. Notably, this finding appears to be potentially sensitive to the inclusion of outliers. Broadly, we surprisingly observed no significant disparities between White and Black/Hispanic individuals despite the existence of substantial inequities between these groups in terms of broader public health.

### Limitations

There are a number of limitations underlying this study that must be considered when interpreting the results. First and foremost, there is a substantial lack of information on genetic conditions for Native American populations (22). As this population has been neglected substantially in the literature, there are unclear implications surrounding the interpretation of inequities in their exposure to newborn screening policies. Additionally, the dynamic nature of state-level policies for newborn screenings means that the results of our analysis, while valid for 2023, may not be valid in the future. This is particularly important to acknowledge given the discussion of further expansion of the NBS to include full genome testing, which introduces equity concerns regarding the distribution of states that choose to expand in this way and how results are conveyed (23). Additionally, while our analysis looked at state-level policies for newborn screenings, there are other ways in which racial and socioeconomic inequity in newborn screening may manifest, such as access to culturally sensitive follow-up care and genetic counseling. For instance, inequalities in trust in healthcare providers are frequently implicated in racial inequities in health (24). Finally, the detection of systemic inequalities in NBS policies by race and socioeconomic status may be underpowered as a result of the small number of units (number of states) in the analysis.

### Comparison with the literature

To the best of our knowledge, no other study has explored racial or socioeconomic variation in the distribution of NBS policies by state. While universal testing is always better, our results contrast with past literature that posits inequality in newborn screening policies as a potential source of racial disparities in infant and child health (25). Other past research suggests mixed evidence regarding the impact of NBS policies on infant health equity (26). Ultimately, while racial and socioeconomic inequalities in infant and child health exist in the United States, our research, which has mixed agreement with conclusions of past work, suggests that inter-state inequality in newborn screening policies are likely not responsible for this variation.

### Implications

There is a stringent, and frequently lengthy, decision-making process for The Advisory Committee to add to the list of RUSP (27). With genetic technologies rapidly advancing, access to genomic information will become increasingly beneficial as more precision medicine techniques become available. Genomic testing as part of NBS has the potential to improve health equity on a large scale, but the time it takes for conditions to get added to the RUSP may mean that therapies and treatments could be available without equal access to care. However, the implications of genomic testing successfully being added to NBS also shine light on the lack of healthcare providers available for proper genetic health information education. The scale at which the population would have access to genetic information would certainly increase the number of individuals and families seeking help for the interpretation of results and is likely to exacerbate existing healthcare access disparities (28).

Ultimately, a central implication of this study is that future research must focus on investigating other mechanisms by which racial and socioeconomic inequities in infant health may manifest. This research suggests that state-level policies are likely not a major source of disparities currently, but it is an important comparison to continue to analyze as states choose to expand their NBS policies. While healthcare provider trust has been studied extensively in the broader healthcare literature, it has not been as extensively studied in the genetic counselor context, which NBS expansion for more genetic conditions and genomic sequencing may reveal as a need. In fact, Ersig et al. suggest that our healthcare system may not be equipped well enough to properly address the psychosocial and ethical concerns of genomic NBS currently (2023). Sobotka & Ross notes that genomic NBS expansion has been specifically discussed for genes associated with neurodevelopmental disorders, in which a diagnosis requires greater access to healthcare and therapies that could worsen disparities (2023) (29). NBS policies will also face ethical concerns as genomic research progresses, and the public must grapple with testing consent and privacy (30). Future research that explores racial and socioeconomic inequity in genetic counselor-patient relationships may shed further light on racial and socioeconomic inequities in newborn screenings.

## Data availability statement

Publicly available datasets were analyzed in this study. This data can be found here: socialexplorer.com.

## Author contributions

SB: Conceptualization, Data curation, Project administration, Supervision, Writing – original draft, Writing – review & editing. KV: Conceptualization, Data curation, Formal analysis, Methodology, Writing – original draft, Writing – review & editing.
